# The experience of loneliness among the Chinese bereaved parents—a qualitative study from the life course perspective

**DOI:** 10.1186/s12877-023-03865-7

**Published:** 2023-03-20

**Authors:** Qian Hu, Ning Wang

**Affiliations:** 1 Department of Sociology, School of Public Administration, Guangzhou University, Guangzhou, China; 2 School of Social and Public Administration, East China University of Science and Technology, Shanghai, China

**Keywords:** *Shidu*, Bereavement, Loneliness, Life course perspective, Qualitative methods

## Abstract

**Background:**

With the implementation of the 37 years one-child policy, many couples only have one child in China. Chinese parents whose only child died and did not give birth to or adopt another child are known as “*Shidu*” parents or “*Shiduer*”. Characterised by elements of childlessness, bereavement, and ageing, *Shiduer* are at a higher risk of experiencing loneliness. However, little is known about their loneliness experience. Adopting a life course perspective, this research aims to investigate how loneliness was experienced and coped by older Chinese *Shidu* parents and identify the most vulnerable groups for policy intervention.

**Methods:**

Qualitative method was adopted for this study. Semi-structured interviews were conducted with 27 participants from urban and rural Wuhan, the capital city of Hubei province in central China, to collect data on participants’ life course related resources and loneliness experience after bereavement. An abductive approach was used to analyse the data.

**Results:**

The results demonstrate that the social environment (urban/rural), timing of bereavement (midlife/older age), social network (strong/weak), and coping strategies (escape-avoidance/problem-solving) differentiate the experience of loneliness among the *Shiduer*. Those who lived in rural communities, those bereaved in older age, those who had a weak social network, and those who adopted the escape-avoidance strategy were found vulnerable and suffered from more chronic and intensive loneliness than their counterparts without these characteristics.

**Conclusion:**

This study is among the first attempts to examine loneliness experience and coping among older Chinese bereaved parents from a qualitative, life course perspective. It provides insights into how loneliness has been perceived and experienced differently among the bereaved one-child parents in China. The results of the current study provide important implications for policymakers and practitioners/social workers for the intervention of loneliness.

**Supplementary Information:**

The online version contains supplementary material available at 10.1186/s12877-023-03865-7.

## Introduction

Loneliness is an experience that has been suggested to be of high prevalence in later life [1, 2]. Moreover, among older adults, those who outlived their adult children were found to have a higher risk of experiencing loneliness [3]. Although the literature on the relationship between childlessness and loneliness has inconsistent findings [4–6], being childless has been found to have a significant negative effect on loneliness and depression in the Chinese context [7, 8]. Against this background, the current study aims to investigate the loneliness experience among a specific social group in China, called “*Shiduer*”, from the life course perspective [9]. The social group of *Shiduer* is specific for research on loneliness as it combines the elements of childlessness, bereavement, and ageing. A life course perspective is adopted in this study to guide the data analysis and thus achieve a deeper and wider scope of understanding of the loneliness experience.

*Shidu* is the Chinese translation for “losing one’s only child and becoming childless at a post-reproductive age”; it is a culturally-specific and cohort-specific phenomenon which is related to the 37-year implementation of the One-child policy (1979–2016) in China [10]. There is no consensus on the exact number of *Shidu* families, due to different definitions and calculating methods, but the Chinese official estimated that there were around one million *Shidu* families where the mother had passed her reproductive age (defined as being 49 years old) by 2012 and such number was increased by approximately 76,000 households per year [11].Therefore, the study of *Shiduer* is of high policy relevance as the period and cohort effects begin to play out. This study is also significant in terms of adding loneliness experience in Chinese cultural context to the global loneliness literature and providing important implications for studies in other contexts.

There is a considerable amount of literature focusing on loneliness, covering various topics related to its dimensions, measurement, influential factors, consequences, and dynamics over the life course [12–16]. Among the relevant qualitative studies, many have focused on interpretation of the loneliness experience at a single time point, while few have paid attention to the complex influential factors of the loneliness experience from a life course perspective and investigated individuals’ life course dynamics [17–19]. Moreover, studies on life events and transitions that trigger chronic loneliness were relatively scarce [20]. As loneliness is an experience that evolves over time and differs from person to person, it is crucial to adopt a life course perspective and consider the social contexts of life events, timing, linked lives, and human agency in shaping individuals’ experience. This study aims to investigate the loneliness of Chinese *Shiduer* from a qualitative life course perspective.

Loneliness is generally defined as the discrepancy between an individual’s desired and actual interpersonal relationships [21]. More specifically, Weiss [22] identified two types of loneliness: emotional loneliness, which reflects an individual’s perceived lack of intimate attachment; and social loneliness, which is associated with a lack of social engagement in the community or larger society [23]. Another form of loneliness, existential loneliness, has been defined as a primary and inevitable condition of existence, which generally ties to a lack of meaning, a sense of isolation and the associated feelings of helplessness, as well as feelings of “groundlessness” when facing death [24–26].

### Understanding loneliness from a life course perspective

The life course perspective allows researchers to consider loneliness as a dynamic process across the life span in which the intensity and duration thereof may vary [20]. From a longitudinal perspective, loneliness can be classified as transient, situational, chronic, or seasonal [27]. It also helps distinguish those who have always been lonely from those who recently became lonely [28]. Using data from the European Social Survey, Victor and Yang [29] found that the level of loneliness followed a U shape over the life course, peaking before the age of 25 and after the age of 65. This finding indicates that loneliness is to some extent associated with ageing, during which process the numbers of social relationships decrease and social networks reduce [30, 31]. The age-related losses may be due to role transition (e.g., retirement-related), declining health, and increasing mobility limitations [27].

Social and historical context, timing of life events and transitions, linked lives, and human agency are four key concepts in the life course perspective which can help to enhance our understanding of loneliness [32]. The life course perspective acknowledges the impact of social and historical time in shaping the loneliness experience from a macro perspective [33]. Economic condition and state policy at a given period may shift the demographic patterns, leading to the changing contexts for social relationship [34]. This may influence different birth cohorts to follow specific pathways to loneliness. For example, the economic reform and large-scale rural to urban migration trend have resulted in a high proportion of empty nest older people in rural China; this group has been found to be more prone to loneliness than older cohorts who co-reside with their adult children [35]. Similarly, China’s One-child policy launched in the late 1970s and abolished in 2016 generally influenced the family formation of the urban 1950s and later cohorts by restricting fertility to one child per family [36]. The decreased family size established an important background for the later life kinship scenarios of the cohorts that were influenced, which may affect the emotional aspect of older people’s loneliness. On the other hand, the characteristics of some social context and general cultural values may also make people vulnerable to loneliness. Such factors are regarded as predisposing factors for loneliness [37].

Personal life events and their specific timings may influence the intensity of the loneliness experience. Many of the life events and transitions can be precipitating factors that trigger the onset of loneliness by creating the mismatch between a person’s actual social relations and his/her desired ones [38]. Transitions such as becoming a parent, becoming a carer, divorce, retirement, and bereavement were found related to loneliness experience [20]. Moreover, the assigned meaning, lived consequences, and management of the life events may vary according to different timings of life events [39]. For example, divorce and bereavement have been suggested to pose threats to valued interpersonal relationships, and are associated with feelings of loneliness [13]. However, experiencing divorce and bereavement (either parental, spousal, or adult child bereavement) in later life may incur more intense feelings of loneliness, as coping strategies for loss become more limited with ageing [28, 40].

Human lives are embedded in social relationships with kin and friends across the life course [32]. Spouse/partner, children, family and friends are the main “linked lives” of individuals; support from each of the relational sources alleviates loneliness, while strain from all four sources intensifies loneliness [41]. Deficits in relationships, such as lack of closeness with friends, lack of meaningful interactions and relatively small networks may associate with loneliness feelings [42]. Research on the changing dynamics of the “linked lives” of individuals, both in their family and broader social networks, can facilitate understanding of issues related to the individuals’ loneliness experience [42].

Human agency implies that an individual can act intentionally to relieve their loneliness. Human agency relates to resilience, which is defined as effectively negotiating, adapting to, or managing significant sources of stress or trauma [43]. Research has suggested that resilience traits such as self-efficacy in the area of establishing and maintaining social contacts were associated with stress resistance in face of loneliness [44]. Human agency is dependent on interpersonal relations and older people were found capable to actively cope with loneliness by maintaining regular contacts with friends and family [45]. Human agency is also influenced by social structure which determines the opportunities for agentic actions [46].

### Loneliness among Chinese *Shidu* parents

This study focuses on Chinese *Shidu* parents who were bereaved by the death of their only child and became childless at post-reproductive age (49 and over). This selection criteria are in accordance with the official definition of Chinese *Shiduer* who are entitled to formal social support [47]. The phenomenon of *Shiduer* has been widely reported by the mass media in recent years due to its rapidly increasing number, particularly since the first generation of parents from one-child families entered older age [48, 49]. Existing quantitative studies have found that *Shiduer* have worse physical and mental health than non-bereaved parents [50–52]. The qualitative studies, on the other hand, have generally corroborated the quantitative findings regarding the deteriorating physical and mental health, reduced social networks and participation, loss of meaning in life, and lack of care and security of *Shiduer* [9, 10, 53, 54].

In Chinese context, the *Shiduer* may face specific social stigma as losing the only child and being childless in later life entails a failure to the familism custom and an evil fate [10, 54]. Childlessness is seen as a failure to carry on the family line and being extremely non-filial to the ancestors in Confucianism, which will cause enormous psychological burden and low self-esteem among *Shiduer* [54]. In addition, losing one’s only child and becoming childless in later life is regarded as a worst divine retribution, implying that the person has done bad things in the previous life. This belief often results in a severe social stigma that may lead to social discrimination and estrangement for *Shiduer* in their social networks [10]. Moreover, some *Shiduer* have internalised the social stigma and become self-isolated, and some only interact with their self-support network called *Tongmingren (the translation for people who share the same destiny)*. The above findings suggest that the *Shiduer* are a group at a higher risk of loneliness [55]. To our knowledge, however, no study has investigated the dynamics of loneliness experienced by *Shiduer* and the implications for targeted intervention from a qualitative life course perspective. The current study aims to fill this gap.

## Methods

This research adopted a qualitative interpretive approach and an in-depth interview method to explore *Shidu* parents’ experiences of loneliness. Unlike focus groups and qualitative surveys, qualitative interviews were more flexible and ideally suited to experience-type research questions and topics in which participants have a personal stake [56, 57]. Such an approach is appropriate for this study as it allows participants some flexibility and privacy to share their loneliness experience to a researcher at the time and place that they feel comfortable.

### Sampling and data collection

We adopted both purposive sampling and theoretical sampling in this study. Purposive sampling was used at the beginning with defined criteria for recruiting those *Shiduer* who had reported loneliness experience during our previous studies. Theoretical sampling was applied after the initial analysis of the data, when we wanted to involve more participants from particular groups or different locations to provide contrast with existing participants and build extra heterogeneity into the sample [58].

Loneliness experience has been identified as a sensitive topic that participants may feel difficult to admit and talk with strangers [59], therefore, we decided to start recruiting participants from our previous research networks where mutual trust and rapport had been achieved between the researchers and participants [45]. In the first stage, purposive sampling was adopted to recruit participants in the research network who: 1) were aged 49 and over at the time of the study to be in line with the official recognition for Chinese *Shiduer*; 2) had self-reported any loneliness experience during our previous studies; and 3) were cognitively capable to do a face-to-face interview. Seventeen participants agreed and provided informed consent.

After the initial analysis of the data, we found that our sample was homogeneous: most participants were urban residents, had lost their child for a long time, and were in a state of transient and situational loneliness. That is, they generally experience loneliness when something triggers the feeling, or at special times, such as festivals or the child’s birthday or the anniversary of their death. To capture a wider range of research participants with different characteristics, we designed a second stage sampling and included more *Shiduer* who were from rural areas, and who were more recently bereaved. Participants were accessed via the snowballing method. At this stage, trust was built through the detailed introduction of the research aim by intermediaries. Ten more *Shiduer* agreed to participate and signed informed consent forms. A final sample of 27 *Shidu* parents participated in this research and data saturation was reached as no new themes had emerged since the 23rd interview.

The interviews were mainly focused around the topic of *Shiduers*’ previous life trajectories and loneliness experience after their only child’s death. The interview schedule initially included the participants’ demographic and socioeconomic information. We then collected the following information: a biographical narrative about their bereavement and grief trajectory; a brief life history; kinship and social networks before and after bereavement; experience of and reactions to the feeling of loneliness (depending on participants’ own definition and understanding). All interviews were conducted by the first author in quiet restaurants or participants’ own homes between March and June 2019 and were audio-recorded. Interviews ranged in length from 61–123 min, with a mean of 82 min.

### Research participants

All 27 participants were recruited from urban and rural areas of Wuhan. Their demographic and socioeconomic characteristics, as well as information on bereavement are shown below (see Table 1; for detailed information, please see Supplementary material).

**Table 1:** Participants’ characteristics

	Category	N	%
Gender	Female	15	55.6%
	Male	12	44.4%
Living region	Urban	20	74.1%
	Rural	7	25.9%
Current age	49–59	6	22.2%
	60–69	15	55.6%
	70 +	6	22.2%
Age at bereavement	49-	6	22.2%
	50–59	14	51.9%
	60 +	7	25.9%
Time since bereavement	1–5 years	4	14.8%
	6–10 years	12	44.4%
	11–15 years	9	33.3%
	16 years and over	2	7.4%
Educational attainment	Primary school and below	7	25.9%
	Junior high school	12	44.4%
	Senior high school	7	25.9%
	College and above	1	3.7%
Marital status	Married	19	70.4%
	Divorced	3	11.1%
	Widowed	5	18.5%
Living arrangement	Living with spouse	19	70.4%
	Living with relatives/friends	1	3.7%
	Living alone	7	25.9%
Self-assessment of economic status	Better than average	6	22.2%
	Average	12	44.4%
	Worse than average	9	33.3%
Self-reported health status	Good	5	18.5%
	Average	8	29.6%
	Poor	14	51.9%
Self-assessment of supportive social networks	Rich	7	25.9%
	Average	4	14.8%
	Poor	16	59.3%

### Data analysis

The analytical approach is abductive including a direct content analysis using the life course framework and an inductive open coding to the interview transcripts [60].

The data analysis comprised six steps. First, both authors read the transcripts several times to familiarise themselves with the data. Second, an initial coding framework was constructed based on the four key themes from the life course paradigm (social and historical background, timing, linked lives, and human agency). Third, both authors assigned inductive open coding to each transcript separately, and categorised the codes to the initial coding structure. Codes that did not fit in the initial coding structure were assigned to new themes. The above steps had applied to data collected from two phases of sampling. Then, fourth, both authors discussed the existing coding and confirmed the final coding structure. Fifth, by reading the coding and transcripts several times, the two authors entered discussion to identify the most representative cases and quotations to illustrate the themes. Finally, we crosschecked and modified our results by sending them to five research participants who had reported that they would be willing to provide feedback on the results. Three participants made amendments to the theme structure before agreeing on the final themes. As we focus on the loneliness experience from a life course perspective in this article, we only report the themes that relate to this perspective and have discarded the scattered themes that were of no relevance to this perspective in the result section.

This study was granted ethical approval from the research institution of the first author. Confidentiality and anonymity of the data were strictly complied. During the research process, reflexive notes and regular team discussion were used to reduce potential interpretation bias.

## Results

### Social environment

The life course perspective proposes that human lives are embedded in social and historical time and space, and are influenced by social and cultural environments [61]. In our research, the participants were born in the 1940s, 1950s, and 1960s, and, as such, they had experienced different social and historical events over the course of their lives. However, with respect to the results related to the experience of loneliness, we did not find any significant cohort or period effects. The urban/rural residence, on the other hand, were found to be an important predisposing factor that influence participants’ loneliness experience. The results demonstrated that participants from rural areas generally experienced more intensive and longer-term feelings of loneliness than their urban counterparts, and they faced more challenges in their life restoration and life meaning reconstruction. The difference could be explained by two specific factors.

### The influence of social culture

Chinese traditional culture emphases the continuity of family line as a key element for filial piety. Families who fail to have a descendant may face significant social stigma and exclusion and this is especially severe in rural China. In our sample, seven rural *Shidu* parents reported experience of perceived stigma and exclusion from their communities, which significantly contributed to their loneliness experience. Case 4 said:

I have lived here for more than 60 years. Before my son’s death, I went to play Mah-jong with the villagers every day. However, now I dare not to join them as I know that they have been speaking ill of me. Once when I fought with a neighbour, he verbally abused me and said that I had lost my only son because I had done evil in my previous life. The words were really heart-breaking. My relatives and neighbours thought I was an unlucky person and did not want to talk to me…... I have no one to talk to and nowhere to escape. You could never imagine the loneliness I have been through. No one could understand my feelings. It was not only the grief, but also all the negative words and viciousness from people around me.

Due to improved education of urban citizens, urban culture is more open, protecting privacy and respecting diversities. Therefore, the urban participants for this study generally live in a more relaxed social environment and do not always face that severe social exclusion. Consequently, they did not report similar loneliness experience caused by stigma and exclusion.

### The influence of social space and resources

In addition to social culture, urban and rural China were differentiated by their social space and the associated resources for life restoration after bereavement. Traditionally, rural people are bounded to the land, and they generally live in a place from birth to death. Rural society is an acquaintance society where people are interconnected by kinships and geographic proximity. In a village, people know each other and *Shiduer* do not have much space for privacy if they want to conceal their *Shidu* identity. Urban China, comparatively, is characterised by individualism and mobility, *Shiduer* are able to move to a new residential space where people do not know their past and they can start a new life after bereavement. Case 7 is an urban male aged 66 who lives after six months to one year of acute grief. After bereavement, he moved away from his original neighbourhood and no one in the new community knew his story except for the community staff and social workers. He and his wife had avoided the potential stigma and begun a new life.

The social support for *Shidu* parents is much better in urban areas compared with rural China. I have a pension of 4000 Chinese Yuan per month, and could get some additional support from the community. I also joined the *Shidu* QQ group [the social media group of *Shidu* parents]. Through this, I know many people who have had a similar experience. We meet frequently and we often travel together. All these activities have alleviated my feeling of loneliness, even though I still feel lonely every now and then, especially during the festivals.

Compared with the rural community, Chinese urban society is more open for opportunities. In our study, the urban *Shiduer* also had better access to formal support and economic capacity for social participation. Such structural factors enable their life restoration and social network reconstruction, which considerably relieved their loneliness after bereavement.

### Timing of bereavement

The results demonstrate that individuals’ feelings of loneliness were related to the timing of *Shidu*, i.e., the life stage at which they had lost their only child. We found that compared with those who were bereaved before the age of 50, those who had lost their child at 60 and over experienced higher level and more chronic loneliness and faced more challenges to restore their lives. In this study, under the theme of timing, we had two representative cases who were bereaved at the ages of 45 and 65 to represent being bereaved in middle life and later life respectively.

### Bereaved at 45 and loneliness eased over time

Case 5 (aged 61) and her husband (aged 62) lost their only son in 2003. The boy died at the age of 18 from a sudden disease. The wife and husband were 45 and 46 respectively at the time of his death. After one month’s paid leave, they both returned to work. They tried to have another child during the first three years after the bereavement, but never succeeded. From the interview, we found that the loneliness trajectory of the bereaved mother had three turning points: bereavement, retirement, and another bereavement for her own mother’s death.

During the first month after my child’s death, my sky collapsed. I was so desperate and lonely that no one could pull me out. The only thing in my life was thinking about my child and everything related to him. However, one day I realised that I was still young and had a long way to go. We might have another child. Then, I pushed myself to return to work and work harder to forget the loneliness and grief, although such a feeling cannot be expelled late at night……years after, time healed until my retirement. Because I retired at the age of 55, and my husband retired at 60, I had nearly five years of feeling lonely again. To reduce this bad feeling, I spent more time caring for my mum. However, she died in 2018. This was another big blow to me and the loneliness returned once more.

The *Shiduer* bereaved before 50 had some similar characteristics in their loneliness trajectories. First, the intensive loneliness phase immediately after *Shidu* was relatively shorter than that for older groups, and the mild loneliness phase was longer due to better adaptation. In our sample, most participants in this age group began to reconstruct their lives after six months to one year of acute grief, and spent two to five years returning to a “normal life” with their feelings of loneliness controlled to an occasional level. As they aged, however, and with the experience of life events such as retirement, hospitalisation, and bereavement of parents or spouse, the feeling of situational and chronic loneliness increased. Considering that they had lost the child at a relatively younger age, they had more time to adapt to life without a child and prepare for old age.

### Bereaved at 65, and long-lasting feeling of loneliness

Case 14 was a rural older man aged 73 at the time of the interview who had been bereaved due to his son’s death in 2011, when he was 65. He was an example of passive adaption and had often engaged in suicidal thoughts and behaviours after *Shidu*. In 2017, he lost his wife and his own health deteriorated the next year. The feeling of loneliness peaked once again in his later life.

I had hoped to rely on my son for support in old age, and who could imagine that he would leave even earlier than myself! I was 65 when my son died, and half of my body was in the grave, what could I do? If I had been forty, I might still have hope and moved on, but I was approaching 70, all was done! There is no meaning in my life. I had lived for 70 years and all was in vain…… Of course, I feel very lonely. When she [his wife] was still alive, I had to take care of her and did not have time for anything else. Now, she has also abandoned me and I do not know who I can rely on and talk to.

The participants who were bereaved after 60 were generally not capable of giving birth or adopting another child, and they were comparatively more vulnerable as they faced higher risks of other bereavements, diseases, and hospitalisation. They also tended to lose the supportive resources they had as well as their capacity to create new resources.

The perceived loneliness among parents who had lost their only child in this age period was generally more intensive and chronic. It appeared that parents who lost an only child in later life were more pessimistic, feeling they were powerless to reconstruct their lives, create resources for later life, or avoid further life risks. Experiencing any other adverse life events, particularly the death of the spouse, could lead to more severe feeling of loneliness. The loneliness experience for this group was not only due to bereavement and social culture, but was also associated with the negative aspect of ageing, according to the participants.

### Social network

The life course perspective examines how individuals interact with their family members, other kin, friends, and associates [32]; therefore, the quantity and quality of social networks play an important role in shaping the loneliness trajectories among *Shidu* parents. The results demonstrated that *Shidu* parents with scarce social networks were likely to suffer from longer-term and more intensive feelings of loneliness than those with rich social networks.

### Rich social networks and reduced feelings of loneliness

The accumulation of supportive social relations in one’s early life has significant influence on coping and the subsequent loneliness experience. Family members, wider kinships, and friends were suggested to provide a good source of support during the period of loneliness after bereavement. Seven participants in this study were well supported by the members of their social networks, including their spouses, parents, siblings, wider kin, and friends. Such support not only involved emotional support, but also daily instrumental help.

**Fig. 1:**
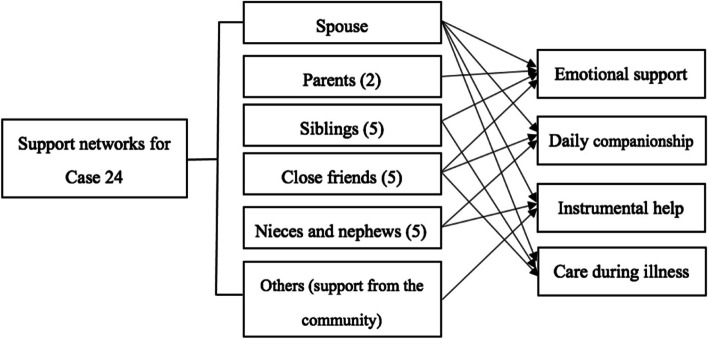
Support from “linked lives” for Case 24

Case 24 (women, aged 64, married) was a representative case with good support network (see Fig. 1). She had a good relationship with her husband, two living parents, and five siblings (two brothers and three sisters); she also had five good friends who were her “safe hands”. The accumulation of supportive social relations in her earlier life meant a lot to her coping after bereavement. During the first three months after her child’s death, she was well supported by her network. She admitted that without such support, she would not have been able to endure the acute grief and endless loneliness due to the child’s death.

The love and support from my relatives and friends were extremely important for me, which made me realise that I was important to many people and provided me with hope and courage……I am the youngest among my siblings, and they all love me throughout my life; we are very close, always…… During the first few months after my son’s death, my sisters took turns living with me to alleviate my grief and loneliness. In every special festival, my siblings, nieces, and nephews came to stay with us. My friends often invited me to travelling. Although I had lost my own child, I was well-supported by people around me, without whom I would not have been able to live through the endless feeling of loneliness...... I sometimes still feel lonely, especially when I am sick and having no kids on call. Friends and relatives cannot accompany you all the time, you know.

In addition to the kin network, we found that self-support groups were also part of the supportive network for *Shidu* parents. Eleven participants in this study reported that communicating with other *Shidu* parents from the self-support groups played an important role in reducing their feelings of loneliness. For example, Case 23 confided:

My child died in 2009. In 2012, I joined a social media group called “*Tongmingren*”. I met many *Shidu* parents through that group and we encouraged and supported each other. We met frequently and travelled together to kill the feeling of loneliness……Last time when I was ill, one *Tongmingren* came to the hospital and cared for me together with my husband. Nevertheless, I still felt lonely during the festivals when families get together. To avoid this feeling, I often travelled together with my *Tongmingren* friends.

However, some *Shiduer* also reported that the function of self-support group in alleviating their loneliness is limited. For example, Case 6 confided:

About six months after my son’s death, I knew a web group of *Shiduer*. People share their heart-broken stories which made me feel that I am not alone. Gradually, we got familiar and started to meet offline. I always join them for dinner and travelling; we talked and cried together, which made me forget the loneliness temporarily. However, people in the group are pessimistic and repeating these kinds of topics, which prevents me from moving on. Later, I felt that I cannot be like this forever, I must push myself to return my normal life.

We found that many *Shiduer* interacted with other *Shidu* parents rather than their previous social networks. It was noteworthy that although *Tongmingren* provided important informal support to each other especially at the early stage after bereavement, only interacting with the *Tongmingren* group could increase the *Shidu* parents’ risk of being socially isolated in the long run [10].

### Restricted support networks and chronic and intensive feelings of loneliness

**Fig. 2:**
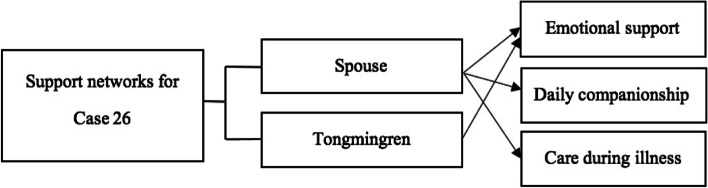
Case 26: weak social network

Compared with Case 24 and Case 23, Case 26 was another extreme. This participant was the owner of a private enterprise, and had good economic resources. However, he did not have strong supportive kinship and other social networks after losing his child (see Fig. 2).

Previously, we [he and his relatives] were not very close; my relatives and friends always tried to borrow money from me, which made me feel disgusted. Because of this, I do not contact them very much. After my child’s death, they all sat on the sidelines and mocked me. My mother stated that she would not leave me any inheritance as I have no successors. My siblings even coveted my own estate. My brother refused to invite me to my nephews’ weddings as they considered me to be someone “unlucky” ……Who else could I count on? I kept moving house and changing my phone number and I stopped contacting my relatives and previous friends……I only contact with some *Tongmingren* friends, we used to hang out and have dinner together. Recently we do not meet as much, because you know, it is difficult to go out and socialize as we are ageing……Yes, I must admit that I am very lonely. It was only me and my wife at home, day by day; it was only us, sitting silently and watching each other. The feeling of loneliness was especially strong and unmanageable when it came to festivals.

The results revealed that the quality of *Shidu* parents’ social networks made a huge difference in their loneliness experience. Those with weak and unsupportive social networks tended to withdraw from social participation and became socially isolated.

### Coping strategies

We found all participants had experienced acute grief and a high level of loneliness immediately after their child’s death; however, after the early period, the way they perceived their loss and the strategies they adapted to life were diverse. Consequently, their loneliness experience varied based on personal choices and actions. In our study, some parents still cannot accept the truth of their children’s death and they tended to escape from the real-life pressure; consequently, they were found in a state of long-term loneliness, while others tried to move on after one to three years of bereavement, and their feelings of loneliness were substantially relieved due to a range of coping actions.

### Escaping from real-life pressure

We found participants who tended to escape exhibited a series of unhealthy behaviours to numb oneself, as well as actions that led to self-isolation. Case 17 was a representative case. Born in 1955, Case 17 was an introverted man who worked in a factory. He had lost his only son in 2013 when he was 58 years old and got divorced two years later because the couple always blamed each other for the death of their child. He took early retirement as he could not accept the loss and return to work. Within two years, he became addicted to smoking and drinking and refused to contact others. This participant had experienced significant loneliness.

I am 64 years old now, and my son has been dead for six years. I always feel very lonely and I think of him all the time. After all these years, things have not been improved and I have never moved on. To me, there is nothing meaningful in life now…...I always felt that I was inferior to others. I seldom leave my home except to buy necessities. There is no point in keeping contact with others, as all their happy moments with their children and grandchildren are triggers for my sorrow and grief.

We also found *Shidu* parents in this group had some features in common. For example, many of them blamed the child’s death on their own mistakes, and generally thought that: “I have no right to live a happy life”, “I am inferior to others”, and “no one can understand my grief”. Regarding behaviours, many of them chose to take early retirement and became self-isolated. They reduced or totally withdrew from normal social interaction and social participation. All these perceptions and behaviours terminated their interactions with other people and society, and consequently, exacerbated their feelings of loneliness.

### Moving on and problem-solving

The results demonstrated that participants who chose to move on, particularly during the early stage of *Shidu*, were relatively better adapted. Regarding their experience of loneliness, many had moved from intensive to occasional feeling of loneliness within a period of one to three years.

Case 19 was an urban married woman who was born in 1958. She owned a real estate company before her only daughter’s death. During the first six months after the child’s death, she could not accept the truth; she sold her company, and locked herself at home all day. Later, however, she made up her mind to change and things began to improve.

Every *Shidu* family has a distinct sad story; however, no one can save you unless you actively change. For me, I experienced desperate loneliness during the first six months after my daughter’s death. I cried all day and I was on the verge of dying. Suddenly one day, I was enlightened. I need to move on, and continue the life of my deceased child. I tried to kill the feeling of loneliness by pushing myself out of my house and talking to friends and family. They are people that I trust; they all have children, I tried not to be sensitive and be open to the topic of children. Such efforts gave me a relief.

These participants generally had a positive outlook and actively engaged in solving problems. First, regarding perception, this group of *Shiduer* had accepted the fact of child death and they reconstructed their relations with the deceased child. For example, many realised that “my deceased child would hope me to move on and live well”, “my child is accompanying me in another way”, or “I am no different from most people; my child has just left me a bit earlier”. Second, in the aspect of behaviour, *Shidu* parents positively returned to work, developed new interests and hobbies, and found new meanings in life. After a period of acute grief, many of them actively rebuilt their social networks and accepted the support and company from their kin and friends. Such actions made a huge difference to their loneliness trajectories and largely relieved their feelings of loneliness during the period of grief.

From the interviews, we also identified some factors that may relate to individual coping. For example, participants who were older, lower educated and in lower socioeconomic status tended to escape. In addition, extroverts were more engaged in social interactions, so that their loneliness was relatively transient and less acute.

In addition, we found participants’ positive outlook and problem-solving, to some extent, were influenced by their early life experience of setbacks and resilience. Like Case 19 recalled:

I worked in the government section previously and I quitted in 1988 to establish my own factory. You cannot imagine how difficult these days were for me as a woman. But I survived, and I became stronger. I know that I should be responsible for not only myself and my family, but also my staff ……I almost gave up my career after my child’s death, by which time my sky collapsed. It was really hard, you know, but I need to move on and lift myself up.

## Discussion and conclusion

This study has investigated the loneliness experience among Chinese *Shidu* parents, bereaved by the death of their only child, from a life course perspective. The life course perspective incorporates both macro and micro angles and considers early life experience in shaping individuals’ loneliness experience. Through an abductive analytical approach, we found almost all participants had experienced profound existential loneliness as they generally felt lack of meaning after the bereavement of their only child. Moreover, all participants had experienced intensive loneliness at the early stage of grief, but their subsequent experience of emotional and social aspects of loneliness differentiated according to their life course related resources.

### Life course related factors and their influence on the loneliness experience

Utilising Elder Jr’s life course framework [32], we observed that the *Shidu* parents’ loneliness experience was influenced by four important factors, which related to the four key themes of the life course perspective: social environment, timing of bereavement, social networks, and coping strategies. Under the social environment, we found that having rural residential status was associated with more social stigma in relation to *Shidu* and less external support resources such as community care and social and financial support. Constrained by financial difficulties, rural *Shidu* parents also had few opportunities to participate in self-support groups – the *Tongmingren*. Therefore, the feeling of loneliness among rural participants were not only emotional, but also social, due to more severe stigma and social isolation [62, 63].

The second factor was the timing of bereavement. We found that participants who had lost their child in midlife (e.g., before the age of 50) were more likely to take positive actions after the first stage of intensive loneliness [64]. They generally thought that their lives were still long, and they could still have opportunities to restore their lives by, for example, trying to have another child, or accumulating resources for later life. Such timing allows people to employ their positive coping, which to some extent distracts them from their feelings of intensive loneliness in later stages of bereavement. Comparatively, losing the only child in older age (e.g., after 60) was associated with more chronic and intensive feelings of loneliness, as people had less time, capacity, and space to create new resources to compensate for the loss, or to prepare for their later lives [28]. Such feelings of hopelessness and insecurity had become additional triggers for their chronic and intensive experience of loneliness.

The third factor was the supportive networks. Consistent with previous studies, we found that support networks played an important role in loneliness reduction [65]. Close kin and friends played a more fundamental role [66]. The *Tongmingren* group were found to be a source of support in the early stage of bereavement as people with similar experience could talk and share their grief. However, we found that participants who only interacted with the *Tongmingren* group were more likely to be stuck in longer term loneliness in the later stage and become socially isolated. This finding is consistent with some previous studies on the self-support group of *Shidu* parents [10].

Finally, as loneliness is a subjective feeling, human agency matters. In this research, the agentic action is reflected as participants’ coping strategies in face of loneliness. Coping is defined as one’s cognitive and behavioural efforts to manage specific external and/or internal demands that are appraised as taxing or exceeding the person’s resources [67]. Previous research has distinguished two forms of coping, the problem-focused, i.e., improving one’s relationships, and emotion-focused, i.e., lowering one’s expectations about relationships [68]. Ways of coping can be various. According to the Coping Scales proposed by Folkman et al. 1986, ways of coping include eight scales, each representing distinctive coping efforts [69]. Following such framework, participants in our study who adopted positive reappraisal and problem-focused coping strategies, and who were willing to return to a normal social life, were found to experience a shorter period of intensive loneliness, and spent longer time in mild and occasional stages of loneliness [64]. While those who adopted the escape-avoidance coping were more likely to experience longer-term and more intensive feelings of loneliness during grief.

**Fig. 3:**
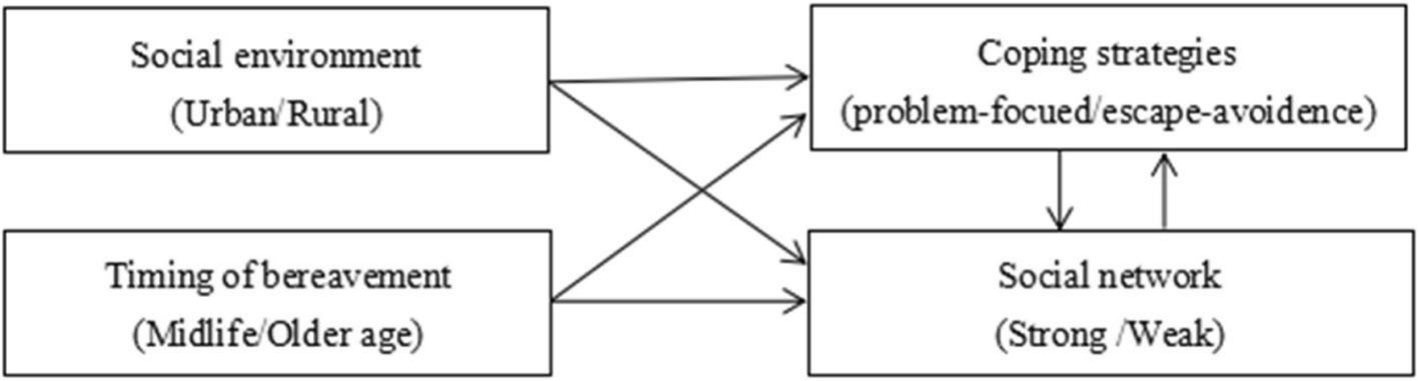
Interactions between life course factors and influences on experience of loneliness

Life course elements often interact to influence individuals’ loneliness experience (see Fig. 3). Larger social structure such as the urban–rural environments and their associated culture, space, and resources, as well as the timing of bereavement and its associated age-specific social capital influence the social networks and opportunity structure that can help one engage with problem-focused coping when trapped in loneliness. While, on the other hand, linked lives and human agency are inseparable; having a supportive social network often help one better adapted to stressful events and positive problem-focused coping will help one alter their troubled interpersonal relations [68, 70].

### Implications for social policy

The results demonstrated that those *Shidu* parents who lived in rural areas, who were bereaved in older age, and who lacked supportive networks and adopted the escape-avoidance coping were more likely to experience longer-term and more intensive feelings of loneliness during grief. These results have important policy implications.

First, policy-makers should aim to reduce social stigma especially in rural areas, and narrow the social support inequality between urban and rural *Shidu* parents. Community education on reducing cultural stigma and improving mutual respect and support should be provided in rural areas. Trained social workers and psychological therapists should be sent regularly to rural communities to provide psychological support for rural *Shidu* parents. Second, policy should support those who were bereaved in midlife to give birth to or adopt another child if they wish to, for example, by financial support for their in-vitro fertilization or lowering the adoption criteria, in order to help them reconstruct life goals. For those bereaved in older age, social support related to ageing and health needs to be enhanced. For example, they should receive regular visits by community staff and social workers, as well as support for hospitalisation and long-term care. Such efforts could help them build up a sense of security in later life despite being childless, and help them to reduce the feelings of helplessness and loneliness. Policy makers should also consider training family members and neighbours on helping *Shidu* parents to restore their social networks, as such factors have been found to be extremely important in reducing feelings of loneliness [71]. The former could be through family social work consultation while the latter could be through community education schemes. Personal initiatives of the *Shiduer* themselves should also be encouraged by policy-makers using professional methods. For example, free consulting services, mindfulness activities and cognitive behavioural therapy could be provided for the *Shidu* parents after bereavement [20]. Although *Shidu* is a context-specific phenomenon, the implications of the current study could also generalise to other societies with familism culture regarding the intention of loneliness for the childless older adults bereaved by their children.

### Limitations

There are also some research limitations. First, partially due to the sensitivity of the topic, we were unable to access those *Shidu* parents who were isolated or who refused to participate. Those people may have different life course resources and thus experience loneliness distinctively. They might lack social connections and critically need intervention. Second, we did not include *Shiduer* who have grandchildren in our analysis, which has restricted the research scope. Future studies may consider extending the scope, and try to incorporate those socially isolated *Shidu* parents and those with grandchildren into the investigation. Furthermore, mixed-methods studies may be considered by future research to combine the qualitative lived experiences with the quantitative evidence in order to further extend our understanding on this issue. Quantitative design should focus on exploring factors that influencing loneliness while qualitative design aims for investigating “how” and “why” in-depth.

## Conclusion

In conclusion, this study explores the loneliness experience among Chinese *Shiduer* using the qualitative approach and from a life course perspective. The findings show that individuals’ life course related elements interact to influence their loneliness experience after bereavement. The social environments and their associated culture, space, and resources, as well as the timing of bereavement and its associated age-specific social capital influence upon people’s social networks and their coping. Moreover, the social networks and coping are inseparable and are fundamentally shaped by individuals’ early life experience. Results show that those who lived in rural communities, those bereaved in older age, those who had a weak social network, and those who adopted the escape-avoidance strategy were vulnerable and suffered from more chronic and intensive loneliness. Based on the empirical findings, specific interventions and policies are advocated.

## Supplementary Information

**Additional file 1.** Supplementary materials

## Data Availability

The datasets used and/or analysed during the current study are available from the corresponding author on reasonable request.
