# Supplementary material for: The experience of loneliness among the Chinese bereaved parents—a qualitative study from the life course perspective
Source: BMC Geriatr. 2023 Mar 20;23:153. doi: 10.1186/s12877-023-03865-7 (PMC10029220; doi:10.1186/s12877-023-03865-7)
Supplement: Supplementary file 1 — Additional file 1. Supplementary materials [file 12877_2023_3865_MOESM1_ESM.docx]

**Supplementary material**

| Case No. | Gender | Living region | Current age | Age at bereavement | Time since bereavement | Educational attainment | Marital status | Living arrangement | Self-assessment of economic status | Self-reported health status | Self-assessment of supportive social networks |
| --- | --- | --- | --- | --- | --- | --- | --- | --- | --- | --- | --- |
| Case 1 | Female | Rural | 56 | 54 | 2 | Senior high school | Married | Living with spouse | Average | Poor | Poor |
| Case 2 | Female | Urban | 76 | 59 | 17 | Primary school and below | Widowed | Living alone | Worse than average | Poor | Poor |
| Case 3 | Male | Urban | 71 | 60 | 11 | Junior high school | Widowed | Living alone | Average | Poor | Poor |
| Case 4 | Male | Rural | 64 | 56 | 8 | Junior high school | Married | Living with spouse | Worse than average | Average | Poor |
| Case 5 | Female | Urban | 61 | 45 | 16 | Senior high school | Married | Living with spouse | Better than average | Good | Average |
| Case 6 | Female | Urban | 57 | 51 | 6 | College and above | Married | Living with spouse | Average | Good | Average |
| Case 7 | Male | Urban | 66 | 57 | 9 | Junior high school | Married | Living with spouse | Better than average | Average | Rich |
| Case 8 | Male | Urban | 62 | 50 | 12 | Senior high school | Married | Living with spouse | Average | Average | Rich |
| Case 9 | Male | Urban | 54 | 52 | 2 | Senior high school | Divorced | Living alone | Average | Good | Poor |
| Case 10 | Female | Urban | 72 | 61 | 11 | Primary school and below | Married | Living with spouse | Worse than average | Poor | Poor |
| Case 11 | Female | Urban | 66 | 65 | 1 | Junior high school | Widowed | Living with relatives/friends | Worse than average | Poor | Poor |
| Case 12 | Female | Urban | 69 | 58 | 11 | Junior high school | Widowed | Living alone | Worse than average | Poor | Poor |
| Case 13 | Male | Rural | 62 | 49 | 13 | Primary school and below | Married | Living with spouse | Worse than average | Average | Average |
| Case 14 | Male | Rural | 73 | 65 | 8 | Primary school and below | Widowed | Living alone | Worse than average | Poor | Poor |
| Case 15 | Female | Urban | 56 | 47 | 9 | Senior high school | Married | Living with spouse | Average | Average | Rich |
| Case 16 | Male | Rural | 63 | 56 | 7 | Junior high school | Married | Living with spouse | Average | Poor | Poor |
| Case 17 | Male | Urban | 64 | 58 | 6 | Junior high school | Divorced | Living alone | Average | Poor | Poor |
| Case 18 | Female | Urban | 56 | 47 | 9 | Senior high school | Married | Living with spouse | Average | Good | Rich |
| Case 19 | Female | Urban | 61 | 53 | 8 | Junior high school | Married | Living with spouse | Better than average | Good | Rich |
| Case 20 | Female | Urban | 71 | 61 | 10 | Junior high school | Married | Living with spouse | Worse than average | Poor | Poor |
| Case 21 | Male | Urban | 52 | 45 | 7 | Senior high school | Married | Living with spouse | Average | Average | Rich |
| Case 22 | Male | Rural | 67 | 55 | 12 | Primary school and below | Married | Living with spouse | Worse than average | Poor | Poor |
| Case 23 | Female | Urban | 69 | 59 | 10 | Junior high school | Married | Living with spouse | Better than average | Average | Average |
| Case 24 | Female | Urban | 64 | 52 | 12 | Junior high school | Married | Living with spouse | Average | Average | Rich |
| Case 25 | Female | Urban | 60 | 47 | 13 | Primary school and below | Divorced | Living alone | Better than average | Poor | Poor |
| Case 26 | Male | Urban | 72 | 61 | 11 | Junior high school | Married | Living with spouse | Better than average | Poor | Poor |
| Case 27 | Female | Rural | 67 | 63 | 4 | Primary school and below | Married | Living with spouse | Average | Poor | Poor |
